# Mr. Walker in Answer to Mr. Woodham

**Published:** 1816-12

**Authors:** 


*For the London Medical and Physical Journal.*

*For the London Medical and Physical Journal.*

[We have taken the liberty of contracting the beginning of Mr. Walker's paper, not that we think it unimportant, but because the question of vaccination is now in the hands of the public, who will, by degrees, form their own judgment.]

Mr. WALKER *in Answer to Mr. Woodham.*

IN my journal of small-pox, which you did me the favour of inserting in a former part of your work, I consider there is a novelty of arrangement, and several other essential points, with regard to the phenomena and treatment, equally useful in practice, and not to be found in the works of Dr. Thomas, or elsewhere.

These premises being admitted, which I consider as incontrovertible, the observation of Mr. Woodham, in his remarks marks on my "Journal of the Progress and Treatment of Small-pox," that it is "a work of supererogation, useless and uncalled for," retorts upon himself, and is referable to the only motive, as it appears to me, by which he has been influenced in making those remarks, viz. an irresistible propensity to the *cacoethes scribendi.*

---

*Remarks on Scirrhus and Cancer;* by the same.

THERE are three different opinions respecting the nature of scirrhus and cancer, considering true genuine scirrhus, as I believe all experienced practitioners are agreed upon, to be the embryo of future cancer.

The first and most common opinion is, that scirrhus, or cancer, is primarily a local affection, of a specific nature, the essence of which is unknown; which, however, in the end, or in its progress, may, and occasionally does, contaminate the system generally. Secondly, by others it is believed, that, wherever an apparently local affection of a scirrhous nature shews itself, whether from previous injury to the part, or arising spontaneously, without being thus to be accounted for, that there has existed in the habit a predisposing cause, inherent in the constitution itself; and that, without this previous disposition in the habit, no kind of injury or incidental circumstance could induce it. And, thirdly, there is an opinion, lately broached, I believe, that the disease consists in a vital principle, possessing existence in itself; in short, that it is an organized parasitical animal, attached to a part of the human body, on which it depredates for its own nourishment, in a manner similar to certain excrescences, so denominated, which exist in the vegetable kingdom.

With respect to these opinions, I shall briefly observe, that, although, in some instances, there may exist a greater disposition in the habit, than in others, for inducing this disease, I am strongly inclined to think, that it would rarely occur without some local exciting cause, operating upon a part, from its peculiar nature or conformation, disposed to take on the scirrhous or cancerous affection; since, although scarcely any part of the human frame is perfectly insusceptible of this affection, we find it, ordinarily speaking, confined to particular parts, of which the breast in women, and the lower lip in men, are by far the most frequent. In either of these instances, and in every other where this disease occurs, it arises probably from the nature of such parts, being less disposed than others for taking on a resolving a resolving or suppurating disposition, together with a peculiar idiosyncrasy of habit, apparently of a peculiarly acrid putrid tendency: hence its more usual occurrence in the decline of life.

With regard to the notion of the disease consisting in a vital principle of itself, as before stated, it appears to be too whimsical to be seriously attended to, especially since it is known that, when the disease is eradicated, by excision, in one part, it sometimes appears in a distant part.

Whatever may be the nature or cause, however, of scirrhus and cancer, one circumstance has hitherto been too evident, viz. that, in order for a cure, extirpation, either by excision or by caustic, the former mode by regular practitioners, and the latter by empirics, ordinarily, or rather constantly, becomes necessary; and this, even in the most recent and favourable cases, is unfortunately not always attended with permanent security, but, according to my observation, generally so.

Such was the state of this matter, according to my own opinion, and, I presume, of every practitioner of adequate experience, when Mr. Young proposed and pursued a mode of cure upon an entirely new principle, as I conceive; which may be briefly stated thus: "By effecting the absorption of the diseased part by compression, in the imitation of what, it is stated, Nature herself does, in an inverse manner, viz. by absorbing a healthy part, when subjected to pressure, by a part in a diseased or unnatural state, as tumours, &c."

There certainly is much of ingenuity and speciousness in the *rationale* of this mode of treatment; and we are informed by the author of various cures having been accomplished by this method, and, in particular, one of the most desperate nature, and in so advanced a state as to render a cure, as it were, next to a miracle, and which Mr. Y. himself had despaired of succeeding in. A circumstantial relation of this case, in which both breasts were affected, and a case of cancer in the lip, are given in the London Medical and Physical Journal, No. 201, pages 410, &c. Some medicines were administered, it should be observed, and some applications used, in the course of the cures, but no great stress is laid upon the effect of these.

Accustomed by a long and uniform observation to the fallacy of the remedies, old and new, proposed for this disease; the relation of the cases alluded to before, nevertheless, attracted somewhat more of my notice, but, I confess, obtained but little of my faith, so incredulous am I become, by uniform disappointment in such matters.

It happened, very lately, that an opportunity offered of my appreciating, by facts, to a certain extent, the merits of this proposed mode of cure.

A lady, somewhat advanced beyond the middle age, whom I knew, with an advanced state of cancer in the breast, was placed under the immediate care of Mr. Young: I was occasionally informed of her supposed amendment, but, ultimately, in the course of some months, I was informed she died in consequence of the disease, whilst under Mr. Young's care.

Secondly, a case presented to me, the progress of which I have attended to, at different periods, from its first appearance, viz. about two years since, to the present time, in a gentlewoman between fifty and sixty, in perfect health, but of a full corpulent habit. This person, as is commonly the case with others, after trying various regular practitioners unavailingly, was led away by the hope of a cure from empirics. This proving fallacious, as ever has and ever will be the case in real cancer, I recommended her, the disease being at this time confined to the breast, to have it taken out, apprizing her of the danger or impropriety of any further delay. At this time she heard of Mr. Young, and placed herself under his immediate care. She remained in London about three weeks, and then returned to Oxford, at his recommendation, with instructions how to pursue his plan. I saw her immediately upon her return, and, on examining the state of the disease, I found it altered evidently for the worse; and there is no question, both from her judgment and my own, injured by the mode of pressure applied; which she declared, and notwithstanding my intreaty to replace herself under his care, alleging the cure of cases, stated by him to have been effected, of a much more deplorable nature than her's, and the precarious event of an operation now, there being a chain, as it were, of carcinomatous knots, running upwards towards the neck, in addition to the former circumscribed state of the breast, and which was now become ulcerated;—she declared she would no longer pursue the plan of Mr. Young, but, under all the unfavourable circumstances represented, prefer the event of extirpation.

Moreover she had learned, and which I have since found to be the fact, that two other persons, with cancerous breasts, in this neighbourhood, have recently experienced, under the care of Mr. Young, the same fate as the lady first mentioned.

Such then is the state, at present, of the effects of this new mode of treatment in cancer, with cases that have occurred here.

The state of these persons, at the time they became

Mr. Young's

Mr. Young's patients, was, unquestionably, extremely unfavourable, but certainly not so hopeless and deplorable as the one stated to have been cured, as before mentioned.

Notwithstanding my assiduous enquiry, I cannot obtain information, at present, of any successful instance, by this mode of treatment, in any person of this neighbourhood. Should it so happen, I shall feel great satisfaction in communicating it, nor do I hear of any case, in a recent, and, of course, in a more favourable state, having been placed under the care of this gentleman.

I should apprehend that lean, or even emaciated, subjects, may be most favourable for this mode of treatment, as well on account of the more easy and effectual application of the due pressure in such cases, as on its effect upon the absorbent system: hence, if this be true, the case I have particularly alluded to as unsuccessful was certainly a peculiarly unfavourable one.

Considering then the cure of scirrhus, or cancer, at this time a desideratum, leaving the proposed method of cure of Mr. Young to be successfully realized, or finally relinquished as ineffective by future experience; and discarding every supposed remedy, both internal and external, which has been hitherto proposed, as chimerical and futile, (extirpation excepted,) I shall proceed to offer briefly what appears to me the only prudent means to be adopted: thus, so soon as the existence of scirrhus in the breast is ascertained, to relinquish entirely, as futile and useless, and loss of time, any attempt at a cure by the specific effect of any internal medicine or application whatever.*

Secondly, to avoid every possible cause of irritation, endeavouring to improve the habit as far as possible, or to ef-

* Whatever may have been asserted respecting the specific effect of Hemlock, exhibited either internally or externally, or conjointly, in this disease, wherever situated, or in any stage of it; I am warranted by the most ample and perfectly satisfactory experience in declaring the entire fallacy of such an opinion. I have witnessed and attended to the effect of this herb for upwards of twenty years, at the Infirmary here, where the annual consumption amounted to several hundred weight, used fresh and dried, under every different modification, and persevered in with the utmost patience, and to the greatest allowable extent with respect to dose, without being able to ascertain any salutary effect whatever from it, which might not have been better obtained by other means. Hemlock *(conium maculatum)* grows plentifully and vigorously in the vicinity of Oxford. I mention this, lest any doubt should arise respecting the identity of the plant.

fect fect an entire change, if possible, by an innocent nutritious regimen. Thirdly, if the case occur in the breast of women, for instance, which, for the most part, does not happen till after the cessation of the menses, to use repeated general and local blood-letting, according to circumstances.* And, fourthly, to attempt an artificial discharge and counter-irritation, effectually kept up, in a part nearest to the disease, and where a cancerous affection, whatever exciting causes might occur, is rarely, if ever, known to show itself, viz. in one or both arms. Lastly, should the scirrhus, notwithstanding these means, increase, threatening open or ulcerated cancer, no time should be lost, but extirpation be immediately resorted to; still, however, persevering in the means already proposed, to prevent or avert a relapse.

I have occasionally observed instances of the occurrence of scirrhus, and consequent cancer, in women past menstruation, after an habitual sore in the leg has become suddenly and permanently well: and, likewise, where a scirrhus-like induration of the breast, where no pregnancy had ever occurred, unexpectedly assumed an imperfect kind of suppuration, the discharge small, and of a serous nature, continuing for a year or more, and the induration in the mean time wasting or dissolving down, and the part remaining a weeping sore. Hence, it should appear in this case, that, from some incidental natural cause, Nature relieved herself in a different, and fortunately less noxious, way. It would be well, if Art could assist Nature in effecting a similar termination in threatening cases.

Although it is advisable, so soon as the nature of the disease is ascertained, especially if it show a disposition to increase, to remove it by extirpation; there is no period at which the disease admits of being removed, that it should be neglected upon account of the danger or probability of its recurrence; since, at any rate, especially in the breast of women, it may be allowable and proper to relieve the person of a mass of disease, although there may be but a slight prospect of an ultimate cure thereby.

Before I became acquainted with Mr. Young's new mode of cure for cancer, by pressure, the following case presented itself to me.

An elderly person, of a full habit, applied to me for the re-

* Care should be taken in the application of leeches, (the ordinary mode of local blood-letting in these cases,) that they be not applied upon, or too near, the indurated or inflamed surface of the diseased part, when thus advanced; as the wounds from the leeches, under such circumstances, are apt to become cancinomatous sores.

moval of a small pendulous tumor, situated upon the inner part of the fore-arm, which had been coming, as he told me, for upwards of thirty years; and had become of late a great annoyance to him, from the violent pain it occasioned in his arm.

My intention was to remove it by ligature, and destroy its base by caustic, this, however, produced such a degree of irritation, that I was compelled to desist. It began to grow again, I then dressed it with dry lint and adhesive plaster, using pressure; by which it was gradually absorbed, and became level with the surrounding skin, and at length healed over. It is still necessary, however, to continue the pressure, to repress its growth. The root of it apparently enters so deep into the flesh, as, in this part, to preclude any attempt at extirpation, but he is freed from any considerable annoyance now from it.

A curious circumstance occurred to myself some time since. By the continued habit, for years, of feeling the point of a pin as a safeguard to the patient, in the application of rollers, particularly such as were damp from embrocation, a callous formed, and at length a warty excrescence rose, with an acute, burning, darting pain in it, which at length alarmed me. I endeavoured to extract it by means of a lancet and forceps, but I found its root penetrated so deep, that I had not resolution to follow it by the lancet, nor could I pull it out by means of the forceps. I showed it to another professional person, who was for cutting it out. Considering the matter in a serious point of view, I adopted the following method: I kept it constantly pared down, in the manner as I have been in the habit of curing deep-rooted corns, covering it by a mild dressing, when, in the course of five or six weeks, it was cured. Nature, as it were, by this assistance, gradually pushing the disease outwards, towards the surface; whereas, before it was uniformly penetrating deeper; although I had long forborne receiving the point of a pin upon it. I have since cured two smaller ones in the same manner.

*Oxford; October 9, 1816.*

---

We have been anxious, ever since Mr. Young's discovery, and the approbation of the late Dr. Denman, to learn from some sources the success of treatment by pressure. Though we have thought it right to admit Mr. Walker's paper, we are far from considering his facts as subversive of the new practice. On the contrary, they admit that relief has been obtained, which is no small consideration in that dreadful disease; and it should be observed, that new remedies are usually assigned to the most desperate forms of disease. We subjoin the following remarks from America.—Edit.

"Mr.

"Mr. Young of England, who, in 1805, published a work on the cure of cancer by natural methods; published also, in 1815, under the auspices of the late Mr. Whitbread, M.P. an account of a second method of curing cancer, by means of pressure. His cases, which are in general those of scirrhus cancer, appear related with candor and authenticity; and certainly seem very extraordinary, and especially the last of them. He operated thus by pressure principally on the female breast; to which it was easy to apply an extensive bandage. The pressure was not only from the first very tight, and in subsequent dressings made as tight as possible, but compresses, and after a time even plates of metal, were placed over the diseased parts, and bound down upon them with the utmost firmness by means of the bandages. The pressure, in consequence of the bandages acting uniformly round the chest, (of which the form is cylindrical,) was easily supported; and the first result was instant ease; which was followed by a gradual alleviation of all the symptoms; medicine in general supplying Mr. Young in these cases with but a subordinate aid.

"Mr. Young pretends to no theory: and, perhaps, he only resolved, by way of experiment, to treat cancer as a common ulcer. Three remarks, however, occur as to the mode in which bandaging is useful in cases of scirrhus cancer. First, the pain seems to disappear in consequence of the torpor produced by the severe pressure applied to the nerves of the part; a principle, of which some surgeons (as M. Larrey) appear to have been sensible. Secondly, since the circulation of the blood-vessels is also impeded by this pressure; these tumors necessarily diminish from the want of nutrition. Thirdly, the absorbents at the same time are rendered peculiarly active; their first law being to have their energy increased under a certain degree of pressure; and their second, that of operating more forcibly on recent and factitious organizations, than on such as are original and natural.

"We say nothing of gelatinous matter in the case of scirrhus cancer; for, though some such matter is frequently seen in these cases when at their maturity; yet bone, cartilage, fat, and membranes, also appear; and a theory is to be desired which shall account for the dispersion of the whole, under the operation of bandages.*

"A proverbial obscurity still envelopes the subject of cancer. To those instances of what has been called cancer, in which the disease from the first produces a loss of substance in the parts, rather than an enlargement; bandaging is, perhaps, not suitable. The same, of course, is still more true of cancers so situated, as not to admit of the application of bandages.—For a double reason, therefore, cancer in the uterus, or the disease bearing that name,

"* As to the component parts of scirrhus cancer, consult here Dr. Adams's pamphlet on Cancerous Breasts; for, though his explanations may not be accepted, his facts are well worthy of attention."

will not admit of it; first, as being inaccessible to the operation; and next, as it is said, that this disease is, perhaps, from its commencement more commonly corrosive, than generated out of a tumour."

---

### COLLECTANEA MEDICA,

